# Supplementary material for: Tuning starch granule size distributions in durum wheat using genetic variation at a single locus
Source: Theor Appl Genet. 2025 Aug 25;138(9):227. doi: 10.1007/s00122-025-05013-8 (PMC12379660; doi:10.1007/s00122-025-05013-8)
Supplement: Supplementary file 1 — (DOCX 1498 kb) [file 122_2025_5013_MOESM1_ESM.docx]

**Supplemental Figures**

**
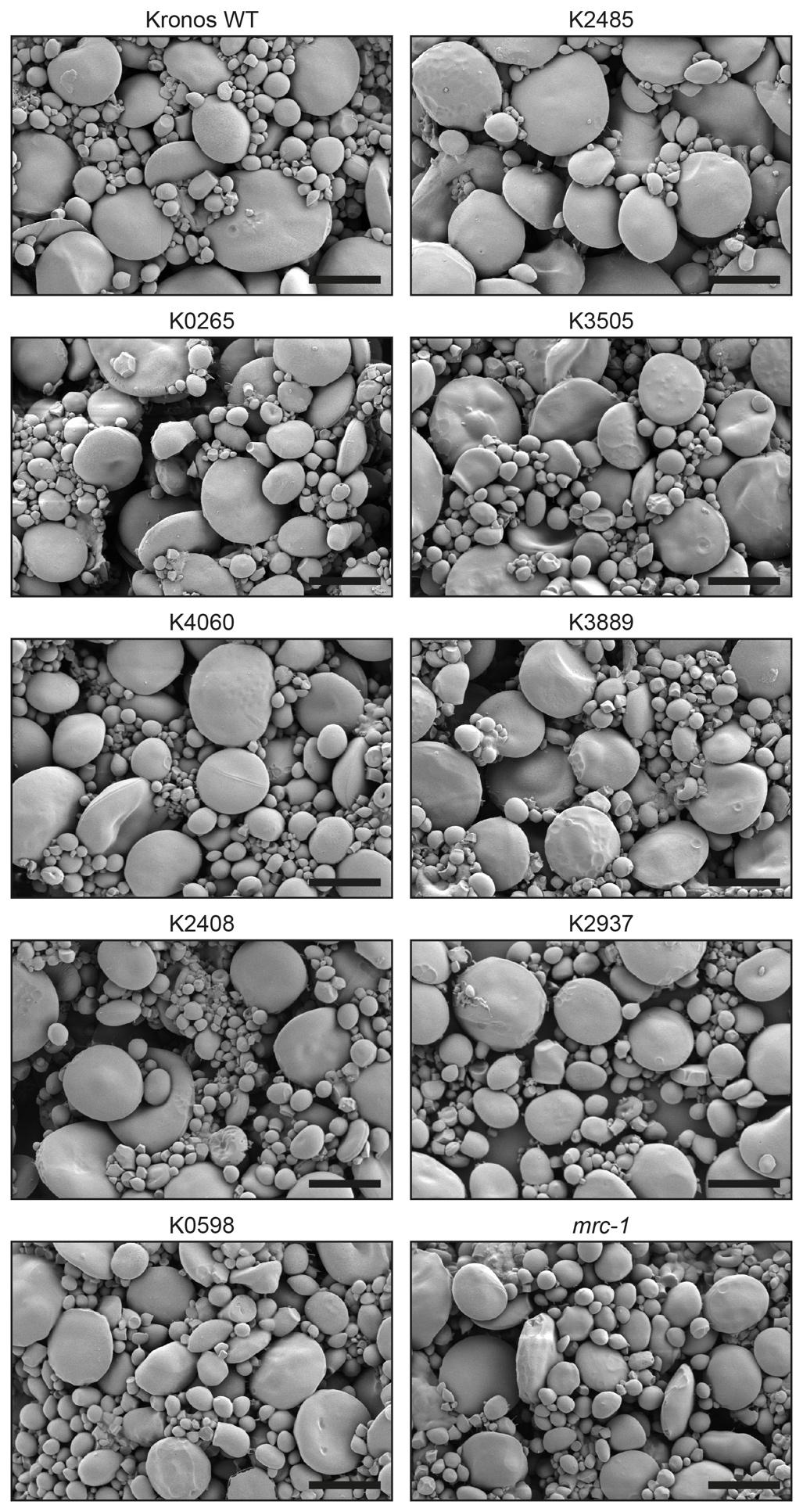
**

**Supplemental Figure 1: Starch granule morphology of Kronos TILLING mutants in Experiment 2.** Purified starch granules were imaged with a Scanning Electron Microscope. Bars = 20 µm.

**
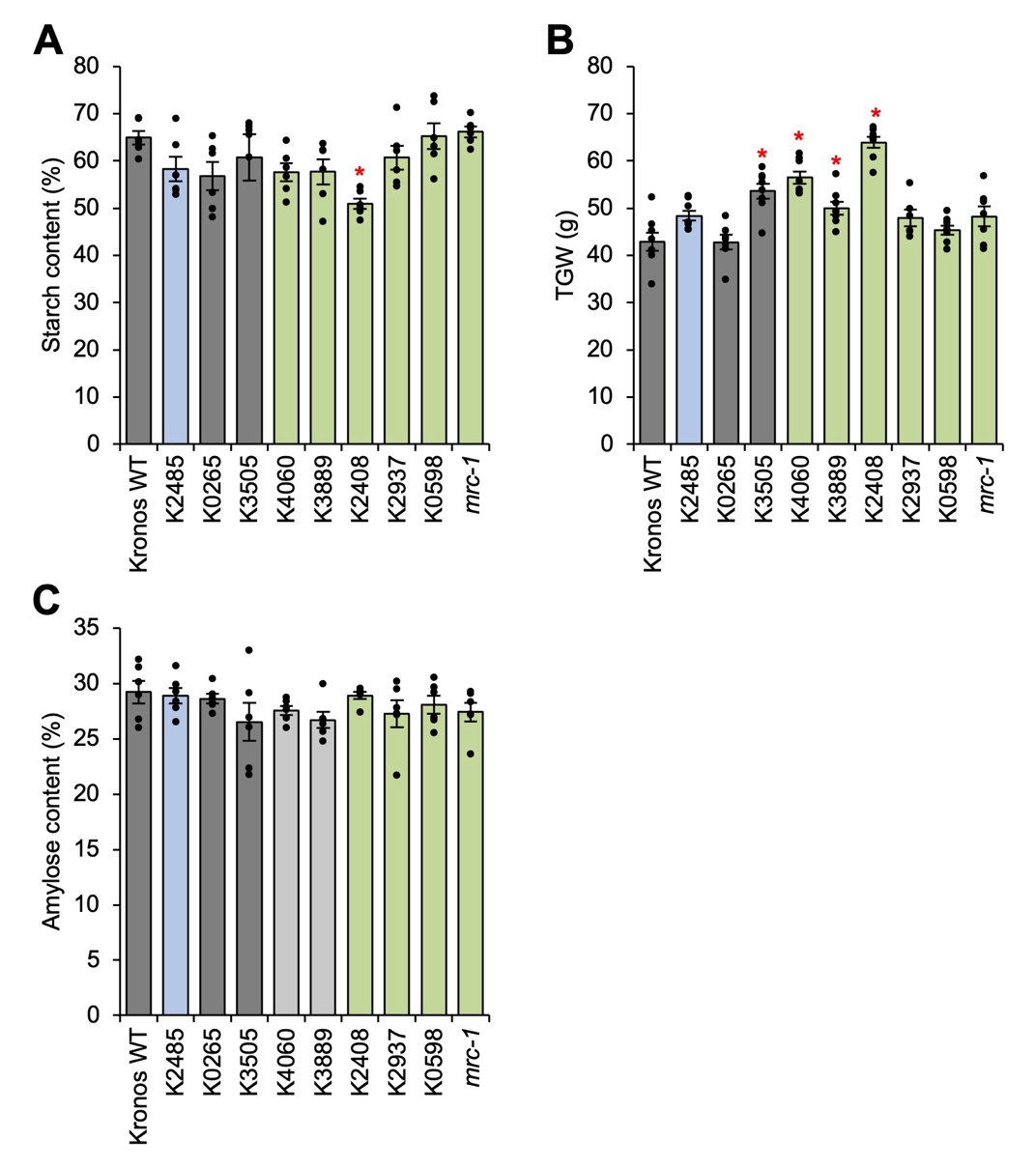
**

**Supplemental Figure 2: Starch content, composition and grain weight of mutants examined in Experiment 2.** **(A)** Total starch content was determined by enzymatic quantification **(B)** Thousand Grain Weight (TGW) was determined using the Marvin seed analyser. **(C)** Amylose content of starch was determined using iodine colourimetry. Values are the mean ± standard error of the mean (SEM) from *n*=6 (panel A and C) or *n*=6-8 (panel B) biological replicates, where each replicate (shown as an individual data point) was prepared from grains harvested from a separate plant. Values marked with an asterisk are significantly different to the Kronos wild type (WT) under a one-way ANOVA and Tukey’s HSD test (p < 0.05).


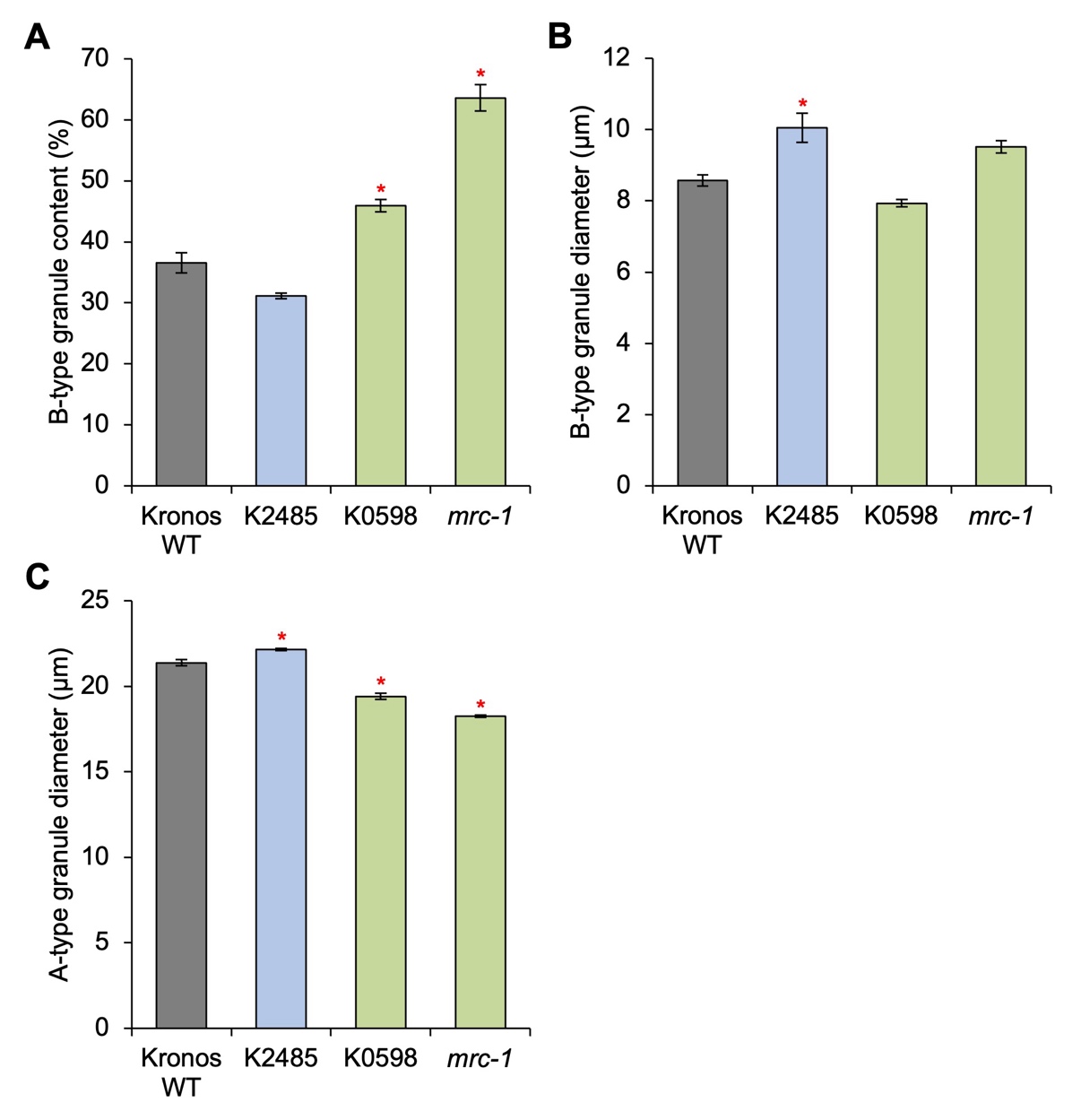


**Supplemental Figure 3: Granule size parameters on large-scale starch extracts used for the Rapid Visco Analysis.** Coulter Counter analyses were carried out on starches (shown in Figure 3A), and granule size parameters were calculated using curve-fitting analyses. **(A)** B-type granule content (by relative volume). **(B)** Mean B-type granule diameter. **(C)** Mean A-type granule diameter. Values are the mean ± standard error of the mean (SEM) from *n* = 3 replicate measurements, and those marked with an asterisk are significantly different to the Kronos wild type (WT) under a one-way ANOVA and Tukey’s HSD test (p < 0.05).


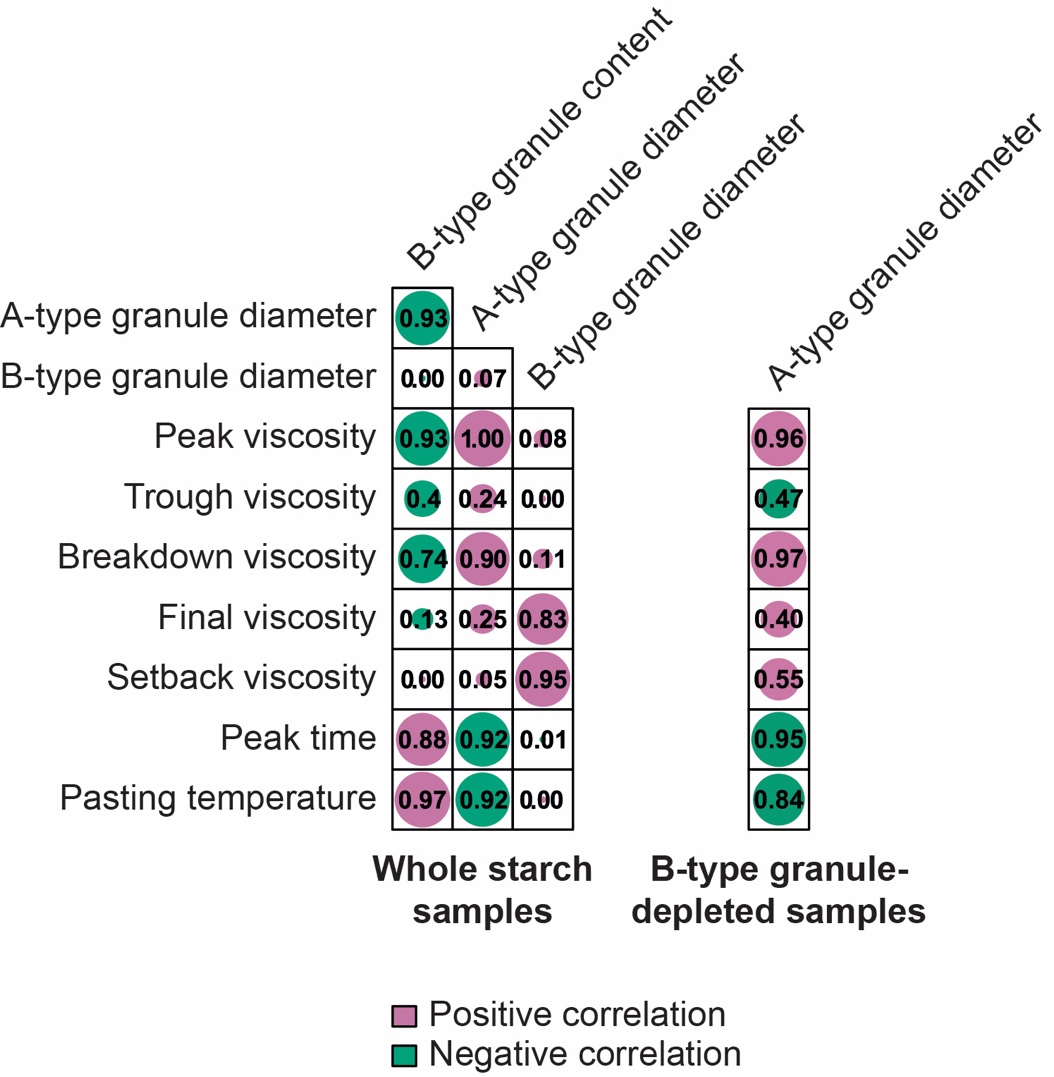


**Supplemental Figure 4: Plot of R^2^ values between pairwise linear regressions for granule size and pasting parameters.** Numbers represent the R^2^ value, while the size of the dot is proportional to the value. Positive correlations are marked with pink dots, while negative correlations are marked with green dots.


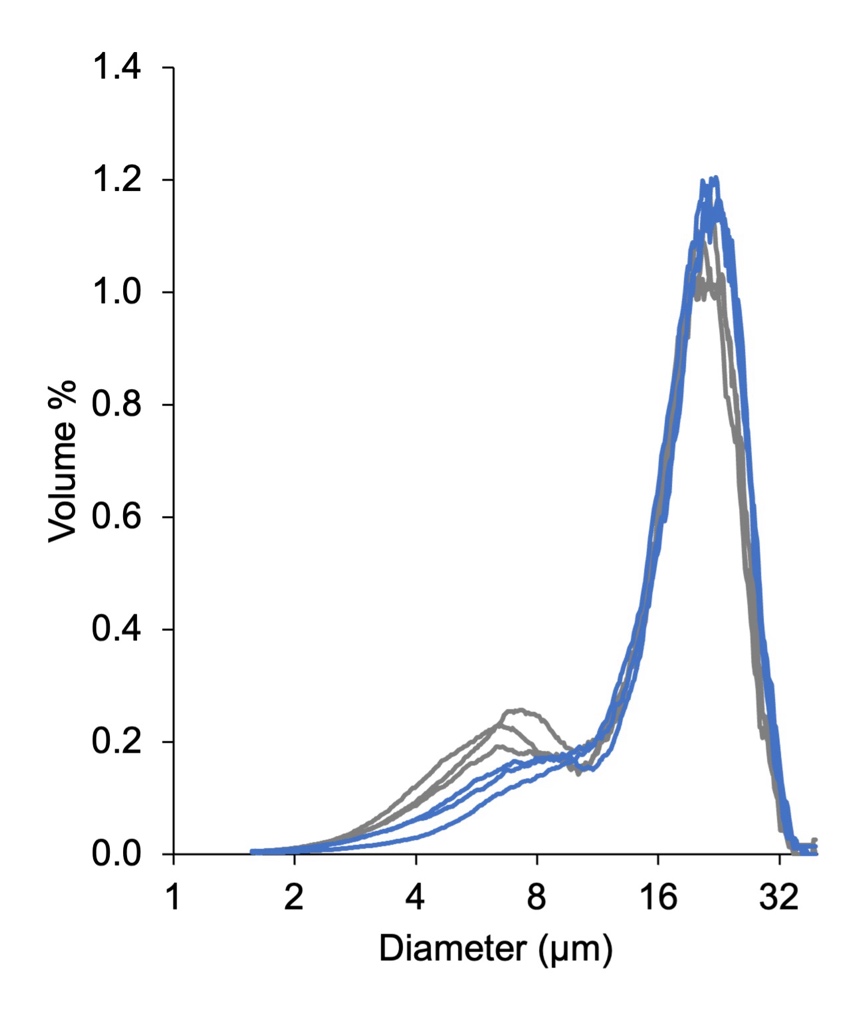


**Supplemental Figure 5: The low B-type granule phenotype in K2485 is maintained after backcrossing.** The K2485 line analysed in Experiments 1 and 2 was backcrossed once to the Kronos WT. In the F_2_ generation, we reisolated three homozygous mutants (blue) and three wild-type segregants (grey). The low B-type granule phenotype segregated with the mutation.

*At*MRC MGFSQAIRLNLASFS-------------SPSPCDYCLTRVVNHKQKSLVAFPSITRRKRH 47

*Os*MRC --------MPPLSPSSSPPATAAAVLRCGSPSCRPVTHELFRQKLSFMVSFQAQHMR--C 50

*Hv*MRC ------MRLSTGCPSPSPAAALAAAHRSTSPSCRTATHVMFRHKLSFMVAFQTQHLK--Y 52

*Tt*MRC-6A ------MRLSIGSPSPSPPPAVAAALRSTPPSRRTASHVMFRQKLSFMEAFQTQHLK--Y 52

*Ta*MRC-6A ------MRLSIGSPSPSPPPAVAAALRSTPPSRRTASHVMFRQKLSFMEAFQTQHLK--Y 52

*Ta*MRC-6D ------MRLSIGSPSPSPPPAVAAALRSTSPSCRTASHVMFRQKLSFMVASQTQHLK--Y 52

: . * :..:* . : : : :

*At*MRC LLLSVQSVLHNTRPNI--NDNGSAESANVLFDKLFARTHRLERQTNQHSVYPDDDDLPYS 105

*Os*MRC APHLIKSVVKGIRANITDGENGATEPARELLERLFAKTQRLDTSASQDSE-------LSM 103

*Hv*MRC APCLIKSVVKSIRSNITDGDNGTTEPARELLERLFAKTQSLDTGASNDSE-------LGV 105

*Tt*MRC-6A APRLIKSVVKGIRSNITDGDNGTTEPARELLERLFARTQSLDTGASHDSE-------LSV 105

*Ta*MRC-6A APRLIKSVVKGIRSNITDGDNGTTEPARELLERLFARTQSLDTGASHDSE-------LSV 105

*Ta*MRC-6D APRLIKSVIKGIRSNITDGDNGTTEPARELLERLFAKTQSLDTGASHDSE-------LSV 105

::**::. * ** .:**::* *. *:::***:*: *: :.:.*

*At*MRC NLGVLESDLEAALVALLKREEDLHDAERKLLSDKNKLNRAKEELEKREKTISEASLKHES 165

*Os*MRC SIDVLKSEFEAALSTLRKKERDLRDAENRVSVDQVRLNRAKKDLDQRERGINRAYARQQE 163

*Hv*MRC SIEVLKSEFEGALSILRKKERDLRNAEKRVSDDRTRLSKTKQDLDQREETIRKVYVRQQD 165

*Tt*MRC-6A SIEVLKSEFEGALSILRNKERDLRSAEKRVSDDRIRLSKTKQDLDQREEAIRKAYVRQQG 165

*Ta*MRC-6A SIEVLKSEFEGALSILRNKERDLRSAEKRVSDDRIRLSKTKQDLDQREEAIRKAYVRQQG 165

*Ta*MRC-6D SIEVLKSEFEGALSILRNKERDLRSAEKRVSDDRIRLSKTKQDLDQREEAIRKAYVRQQG 165

.: **:*::*.** * ::*.**:.**.:: *: :*.::*::*::**. * .. :::

A178

*At*MRC LQEELKRANVELASQAREIEELKHKLRERDEERAALQSSLTLKEEELEKMRQEIANRSKE 225

*Os*MRC MERSLGKASRDLVLQVRQIDNLKLLVDEQDKKIASSQDLLSQKVTEVEKLKQDMLKKNEE 223

*Hv*MRC IEKALKRASRDLALRVKQISNLKLLVEGQDRTIASSQALLSQKVIEVENLKQDMFTKNEE 225

*Tt*MRC-6A IEKALKKASRDLALRVKQISDLKLLVEGQDRTIARSQALLSQKVTEVENLKRDMFKKNEE 225

*Ta*MRC-6A IEKALKKASRDLALRVKQISDLKLLVEGQDRTIARSQALLSQKVTEVENLKRDMFKKNEE 225

*Ta*MRC-6D IEKALKKASRDLALRVKQISNLKLQVEGQDRTIASSQALLSQKVIEVENLKRDMFKKNEE 225

::. * :*. :*. :.::*.:** : :*. * * *: * *:*:::::: .:.:*

R255

*At*MRC VSMAISEFESKSQLLSKANEVVKRQEGEIYALQRALEEKEEELEISKATKKLEQEKLRET 285

*Os*MRC VTLMRSEIKSKEQLLLEANQAAEQQEATIKELRSEIKRKEIDFSRSNELRKANEQKLKIA 283

*Hv*MRC ADLMRSEIKSKEQLLLTANQAVVQQEATVRELQSEIKRKIIDIARSDELRKTNEDKLKVA 285

*Tt*MRC-6A ADLMRSEIRSKEKLLLTANQAIAQQEATVRELQSEIKRKTMDIARSNESRKTNEEKLKVA 285

*Ta*MRC-6A ADLMRSEIRSKEKLLLTANQAIAQQEATVRELQSEIKRKTMDIARSNESRKTNEEKLKVA 285

*Ta*MRC-6D ADLVRSEIRSKEQQLLTANKAIAQQEATVRELQSEIKRKTIDIARSNESRKTNEEKLKVA 285

. : **:.**.: * **:. :**. : *: ::.* :: *. :* :::**: :

L289

*At*MRC EANLKKQTEEWLIAQDEVNKLKEETVKRLGEANETMEDFMKVKKLLTDVRFELISSREAL 345

*Os*MRC EQELERQNMGWLAAQKELKEVAQLACKDMDGIKDTVSDFKRVRSLLDAVRSELIASKEAF 343

*Hv*MRC EQELEKQNLGWLAAQQELKELAQLASDDTDDIKGIITDFKRVRSLLDVVRSELISSKDAF 345

*Tt*MRC-6A EQELEKQSLGWLAAQQELKELAQLAFKDTDDIKGIITDFKRVRSLLDAVRSELISSKDAF 345

*Ta*MRC-6A EQELEKQSLGWLAAQQELKELAQLAFKDTDDIKGIITDFKRVRSLLDAVRSELISSKDAF 345

*Ta*MRC-6D EQELEKQSLGWLAAQQELKELAQLAFKDTDDIKGIITDFKRVRSLLDAVRCELISSKDAF 345

* :*::*. ** **.*:::: : : . . : : ** :*:.** ** ***:*::*:

*At*MRC VFSREQMEEKELLLEKQLEELEEQRKSVLSYMQSLRDAHTEVESERVKLRVVEAKNFALE 405

*Os*MRC SSSRKQIEDQAVQMQKQVQELSGQRLLLSSFNQNLEAARLEIQGKAKELNAAQSRCHELE 403

*Hv*MRC ASSRRQIEDQAVQLREQVQELEDQRVLLMSHTHDLEAARLEIQGKTQELNYAQSRCHELE 405

*Tt*MRC-6A ASSRRQIEDQAVQLQEQVQELEDQRVLLMSYTHDLEAAQLEIQGKTQELNYAQSRCHELE 405

*Ta*MRC-6A ASSRRQIEDQAVQLQEQVQELEDQRVLLMSYTHDLEAAQLEIQGKTQELNYAQSRCHELE 405

*Ta*MRC-6D ASSRRQIEDQAVQLQKQALELEDQQVLLMSYTHDLEAAQLEIQGKTQELKYAQSRCHELE 405

**.*:*:: : :.:* **. *: : *. :.*. *: *::.: :*. .::: . **

*At*MRC REISVQKELLEDLREELQKEKPLLELAMHDISVIQDELYKKANAFQVSQNLLQEKESSLV 465

*Os*MRC SLLLQEKEKVESLEAVLTKERESLEEKTKEVELLQKALVQKENEHSNSLKLVEIKESELL 463

*Hv*MRC SHLLQEMEKVESLEAELTKERQSLEHRTEEVDFLQKELVQKENECTKSQELVKVKEFELL 465

*Tt*MRC-6A SQLLKEMEKVESLEAELTKEKQSLEHRTEEVGFLQKELVQKENECTKSQELVKVKEFELL 465

*Ta*MRC-6A SQLLKEMEKVESLEAELTKEKQSLEHRTEEVGFLQKELVQKENECTKSQELVKVKEFELL 465

*Ta*MRC-6D SQLLQEMEKVESLETELTKERQSLDHRTEEVGFLQKELVRKENECTKSQELVKVKEFELL 465

: : * :*.*. * **: *: .:: .:*. * :* * * :*:: ** .*:

S503

*At*MRC EAKLEIQHLKSEQASLELLLQEKDEELAEARNKLGEVNQEVTELKALMISREDQLMEATE 525

*Os*MRC EARNEVQDMKSKVESIQIAVQEKDSELSETQRRLAEVNSEVVELKQLLDSKEDQLVQVRT 523

*Hv*MRC EARYEVQDMKLKVESIQLAVQEKDSELSATQSRLTEVSSEVVKLQQLLNSKEDQLVQART 525

*Tt*MRC-6A EARQEVQDMKLKVESIQLAVQEKDSELSDTQSRLTEVSSEIVELQQLLNSKKDQLVQART 525

*Ta*MRC-6A EARQEVQDMKLKVESIQLAVQEKDSELSDTQSRLTEVSSEIVELQQLLNSKKDQLVQART 525

*Ta*MRC-6D EARQEVQDMKLKVESIQLAVQEKDSELSDTQSRLTEVSSEIVELQQLLNSKKDQLVQART 525

**: *:*.:* : *::: :****.**: :: :* **..*:.:*: *: *::***::.

D581

*At*MRC MLKEKDVHLHRIEGELGSSKLKVTEAEMVVERIAELTNRLLMSTTNGQNQNAMRINNEIS 585

*Os*MRC ELQDKEQHIQTLQNKLDSMKFSCSQAESVVQKIAELTGNLASSVEGEEMDIYALLDDEIS 583

*Hv*MRC ELHDKEQHIETLESELDSIRLRCSQAESVVQRMAELTGDLASSVKTGETDIYTLLDDEIA 585

*Tt*MRC-6A ELHDKEQHIETLESELDSIRLRCSQAESMVQRMAELTGDLASSVKAGEMDIYTLLDDEIS 585

*Ta*MRC-6A ELHDKEQHIETLESELDSIRLRCSQAESMVQRMAELTGDLASSVKAGEMDIYTLLDDEIS 585

*Ta*MRC-6D ELHDKEQHIETLESELDSIRFRCSQAESMVQRMAELTGDLASSVKAGEMDIYTLLDDEIS 585

*::*: *:. ::.:*.* :: ::** :*:::****. * *. : : :::**:

A625

*At*MRC IDSMQQPLEKPHDDYGMENKRLVMELSFTRENLRMKEMEVLAVQRALTFKDEEINVVMGR 645

*Os*MRC STGT--ALKS----NLHKHNQLEADIEMLKESLHQKDMDLRAAHEALDAKDQELKAVMRR 637

*Hv*MRC SAGT--TLES----NLHKHNQLEADIEMLRECLRHKDMDLRAAHEALDAKDQELKAVLKK 639

*Tt*MRC-6A STST--ALES----NLHKHNQLEADIEMLRECLRHKDMELRAAHEALDAKDQELKAVLKK 639

*Ta*MRC-6A STST--ALES----NLHKHNQLEADIEMLRECLRHKDMELRAAHEALDAKDQELKAVLKK 639

*Ta*MRC-6D STGT--ALES----NLHKHNQLEADIEMLRECLRHKDMDLRAAHEALDAKDQELKAVLKK 639

. *:. ::::* ::.: :* *: *:*:: *.:.** **:*::.*: :

*At*MRC LEAKEQELKKLKEETINDSEDLKVLYALAQERVGEKTMGDLAIEMLQLEAANLEVEAATS 705

*Os*MRC WDVKEEVDKLEGF--LKDPSDIKRPSD---------FSVHMGLQNLQTEAAEVEALAATT 686

*Hv*MRC WDVKERELHELEE--LLDPSATNELACFSNETTEGGVVGEMELQELQIGAAEVEALAATT 697

*Tt*MRC-6A WDVKERELRELEE--LPDPSATNELAGFSSETTEGGIVGEMELPELQIDAAEVEALAATT 697

*Ta*MRC-6A WDVKERELRELEE--LPDPSATNELAGFSSETTEGGIVGEMELPELQIDAAEVEALAATT 697

*Ta*MRC-6D WDVKERELRELEE--LPDPSATNELAVFSSETTEDGIVGEMELPELQIEAAGVEALAATT 697

:.**. : : * . : .: : ** ** :*. ***:

*At*MRC ALQKLAKMSTELLTQADMSIEADTTHT--V----MPERGYSEGSNECLGEVKTEVVRLWS 759

*Os*MRC TLKKLADMAKGFLRSGKTDSGINLVASPSVNSTRIVSKTKPNKEMDMILDAEKEIAGLFS 746

*Hv*MRC ALRKLADMTKDLFKHDKGDSGIDLAASGSQKLRNCDSKMEVHKKTDVILEAEKEITRLFS 757

*Tt*MRC-6A ALRKLADMTKDFFKHVKADSGINLVASESQKIIKCDPKMEVHKKTDVILEAEKEIVRLFS 757

*Ta*MRC-6A ALRKLADMTKDFFKHVKADSGINLVASESQKIIKCDPKMEVHKKTDVILEAEKEIVRLFS 757

*Ta*MRC-6D ALRKLADMTKDFFKHGKADSGIDLVASESQKISKCDPKMEVHKKTDVILEAEKEIVRLFS 757

:*:***.*:. :: . . : . : : . . : : :.:.*:. *:*

*At*MRC LTEKLLENAGIVAGTSTCMEGVIL 783

*Os*MRC LTEQLITEAGIDVAHQA*------ 763

*Hv*MRC LTKQIVTDDIINDVDER*------ 774

*Tt*MRC-6A LTKQIVTDDIINDVEE-------- 773

*Ta*MRC-6A LTKQIVTDDIINDVEE*------- 773

*Ta*MRC-6D LTKQIVTDDTINNLEE*------- 773

**:::: : * .

**Supplemental Figure 6: Amino acid alignment of wheat and barley MRC proteins.** The alignment was conducted using Clustal Omega including MRC sequences from durum wheat (*Tt*MRC-6A, TRITD6Av1G081580.1), bread wheat (*Ta*MRC-6A, TraesCS6A02G180500.1; *Ta*MRC-6D, TraesCS6D02G164600.1), barley (*Hv*MRC, HORVU6Hr1G036020.1), rice (*Os*MRC, LOC_Os02g09340.1) and Arabidopsis (*At*MRC, At4g32190). The positions of the amino acids where mutation increased B-type granule content (A178T, R255K, L289F, S503N and D581N), and the one where mutation decreased B-type granule content (A625T) are highlighted. Green highlight indicates amino acids conserved in all sequences examined, while yellow highlight indicates amino acids that are conserved in all wheat and barley sequences.

| **SUPPLEMENTAL TABLE 1**  KASP markers used in this study | | | | |
| --- | --- | --- | --- | --- |
| Line | Mutation | WT | Mutant | Common |
| **Missense mutants included in the study** | | | | |
| K3745 | A19T | gggtgtgctgcggagagc | gggtgtgctgcggagagt | gttccctctccccatgttcc |
| K3543 | G63S | cgtttgatcaaatcagtcgtaaaag | cgtttgatcaaatcagtcgtaaaaa | ctagctggctcagtcgttcc |
| K1435 | S92N | tgagaagcaccagtgtctaaac | tgagaagcaccagtgtctaaat | gttgctggagcggctatttg |
| K2447 | L149F | tcgcttcctctctctgatcaag | tcgcttcctctctctgatcaaa | agcgcagagaagagggtttc |
| K2937 | A178T | tgaaaaaggcaagtagagatctgg | tgaaaaaggcaagtagagatctga | cccctcaaccagaagcttca |
| K2124 | R196K | ctggttgaggggcaagacag | ctggttgaggggcaagacaa | tcaggtctgcttcctcgttc |
| K2205 | E213K | gaacatatctcgtttgagattttc | gaacatatctcgtttgagattttt | aggggcaagacaggactatt |
| K3499 | R230C | cgcatcaggtctgcttcctc | cgcatcaggtctgcttcctt | tgaggggcaagacaggacta |
| K1431 | E225K | aacgcatcaggtctgcttc | aacgcatcaggtctgcttt | tctcaaacgagatatgttcaagaag |
| K2397 | R230C | tttggacctgatctctgaacg | tttggacctgatctctgaaca | aggggcaagacaggactatt |
| K4060 | R255K | aatttcactctgcagctccc | aatttcactctgcagctcct | gcgttcagagatcaggtcca |
| K0598 | L289F | ccatcctaaactctgcttctcaag | ccatcctaaactctgcttctcaaa | gaagcaacagttagggagct |
| K3533 | A346V | tctatttgtctgcgagaggaag | tctatttgtctgcgagaggaaa | gatgctgtacgctctgaattg |
| K2981 | A356T | tgttcctgcaactgaaccgc | tgttcctgcaactgaaccgt | gatgctgtacgctctgaattg |
| K0775 | L394F | attcaagggaagacacaggagc | attcaagggaagacacaggagt | tcatgacaacgagactgtgca |
| K0265 | M412I | ttgaatcacagttacttaaggaaatg | tgaatcacagttacttaaggaaata | gttctaagctctgtttttctttcgt |
| K3424 | E491K | ggctgttcaagaaaaggattcag | ggctgttcaagaaaaggattcaa | atgtaattcagttctagcctgaac |
| K2408 | S503N | cagagcagactaactgaagtcag | cagagcagactaactgaagtcaa | aggggcaagacaggactatt |
| K1232 | S539N | gccgtatgctatccaactcac | gccgtatgctatccaactcat | ctaaatagcaagaaggatcaactgg |
| K3889 | D581N | tacttgtgcttgaaatttcatcatc | tacttgtgcttgaaatttcatcatt | ctaaatagcaagaaggatcaactgg |
| K3505 | E606K | ccgcaagcattctcttaacatctc | ccgcaagcattctcttaacatctt | acagccctcgagtccaattta |
| K2485 | A625T | cttgatctttggcatcaagtgc | cttgatctttggcatcaagtgt | acagccctcgagtccaattta |
| K4297 | P656L | caagttcatttgtggcactgg | caagttcatttgtggcactga | ggatgtgaaggagagggaac |
| K3416 | S667N | ccgccctctgttgtctcac | ccgccctctgttgtctcat | ggatgtgaaggagagggaac |
| K2348 | E679K | ggcggtattgtaggagagatgg | ggcggtattgtaggagagatga | gcttcgacctccgcagca |
| K2096 | P681S | tgtaggagagatggagctgc | tgtaggagagatggagctgt | gcttcgacctccgcagca |
| K2821 | A687V | gtgcttcgacctccgcag | gtgcttcgacctccgcaa | gtattgtaggagagatggagctg |
| K3666 | D708N | gctttgacgtgtttgaagaaatc | gctttgacgtgtttgaagaaatt | attgtaggagagatggagctg |
| K4193 | S726N | gtattaacttggttgcatcagagag | gtattaacttggttgcatcagagaa | acttccattttaggatcgcatttaa |
| **Missense mutants excluded in the study** | | | | |
| K4533 | A79V | aatggaacgactgagccagc | aatggaacgactgagccagt | gcaccctcgaattcagactt |
| K2450 | A155T | ggaccttgatcagagagaggaag | ggaccttgatcagagagaggaaa | cgcaacgccagatctctact |
| K4618 | R196K | ctggttgaggggcaagacag | ctggttgaggggcaagacaa | tcaggtctgcttcctcgttc |
| K0565 | S329F | agcgtacagcatctagcagag | agcgtacagcatctagcagaa | gggagctgcagagtgaaatt |
| K0665 | A356T | tgttcctgcaactgaaccgc | tgttcctgcaactgaaccgt | gatgctgtacgctctgaattg |
| K3286 | S375F | gaccaaagggtattactgatgtc | gaccaaagggtattactgatgtt | atgacaacgagactgtgcataat |
| K2976 | L409F | tgtcatgaacttgaatcacagttac | tgtcatgaacttgaatcacagttat | gactcgaccttctccatttcctt |
| K1112 | E413K | gcttctagagactcgaccttctc | gcttctagagactcgaccttctt | ggaagacacaggagctcaattat |
| K3145 | E425K | gttctatgttctaagctctgtttttc | gttctatgttctaagctctgtttttt | ctcaattatgcacagtctcgttgt |
| K3657 | T500I | ctgatacacagagcagactaac | ctgatacacagagcagactaat | ctctccagtgtttctatatgttgt |
| K1161 | T525I | atcaactggttcaggctagaac | atcaactggttcaggctagaat | actctccagtgtttctatatgttgt |
| K3661 | D575N | cgtaaaagccggagaaatgg | cgtaaaagccggagaaatga | ggactcgagggctgtact |
| K4205 | L591F | gcttatgtaaattggactcgag | gcttatgtaaattggactcgaa | atggttcaaaggatggctgag |
| K3137 | A603T | cataagcataatcaactggagg | cataagcataatcaactggaga | ttccaactcacgtagttccct |
| K0338 | R700K | ttagtcatatccgcaagcttcc | ttagtcatatccgcaagcttct | gctgccagagcttcaaattgat |
| K4082 | D743N | ctttttcagcttcaagaatcacatc | ctttttcagcttcaagaatcacatt | ttggttgcatcagagagtcaa |
| **Premature stop codon mutants included in the study** | | | | |
| *mrc-1* | W258STOP | agcaacagttagggagctgc | agcaacagttagggagctgt | cctcgattcatttgatctggcg |
| *mrc-2* | W550STOP | catacggctcagatgctcgc | catacggctcagatgctcgt | gcaagatcgccagtgagc |
| All primer sequences are given 5’ to 3’. The wild-type allele primers had the VIC/HEX tail (GAAGGTCGGAGTCAACGGATT) on the 5’ ends, while the mutant allele primers had the FAM tail (GAAGGTGACCAAGTTCATGCT) on the 5’ ends. | | | | |
